# Selective Caries Removal as a Restorative Strategy for Caries Management: Residual Dentin Quality After Chemomechanical and Mechanical Excavation

**DOI:** 10.3390/dj14070419

**Published:** 2026-07-08

**Authors:** Gustavo Gerardo Ramirez-Martinez, Christian Andrea Lopez-Ayuso, Rogelio Jose Scougall-Vilchis, Silvia Rojas-Rueda, Carlos A. Jurado, Abdulrahman Alshabib, Hamid Nurrohman, Rene Garcia-Contreras

**Affiliations:** 1 Interdisciplinary Research Laboratory, Nanostructures and Biomaterials Area, National School of Higher Studies (ENES) Leon Unit, National Autonomous University of Mexico (UNAM), Leon 37684, Mexico; 2 Basic Science Laboratory, Autonomous University of San Luis Potosi, San Luis Potosi 78290, Mexico; 3 Department of Orthodontics, Dental and Advanced Studies Research Center (CIEAO) “Keisaburo Miyata”, Faculty of Dentistry, Autonomous University State of Mexico (UAEMex), Toluca 50130, Mexico; 4 School of Dental Medicine, Ponce Health Sciences University, Ponce, PR 00732, USA; 5 Department of Restorative Dental Science, College of Dentistry, King Saud University, Riyadh 11545, Saudi Arabia; 6 Department of Restorative Dentistry & Prosthodontics, The University of Texas School of Dentistry, Houston, TX 77054, USA

**Keywords:** selective caries removal, minimally invasive dentistry, restorative caries management, residual dentin, chemomechanical caries removal, Vickers microhardness

## Abstract

**Background/Objectives:** Advances in restorative caries management have shifted operative dentistry from complete caries excavation toward selective, tissue-preserving strategies that maintain remineralizable dentin and support adhesive restoration. Chemomechanical systems and self-limiting burs may reduce unnecessary dentin removal, but their effects on residual dentin quality remain relevant to restorative decision-making. This in vitro study compared Carisolv™, Papacarie^®^, Smart Burs™, and conventional tungsten carbide burs with respect to caries removal time, residual bacterial load, laser fluorescence, Vickers microhardness, and scanning electron microscopy (SEM) features of residual dentin. **Methods:** Forty-eight extracted permanent molars were selected. Standardized class I cavities were prepared and subjected to an artificial caries protocol using a demineralizing solution for 240 h. A cariogenic biofilm model was then established using *Streptococcus mutans*. Specimens were allocated to four excavation groups: Carisolv™, Papacarie^®^, Smart Burs™, and conventional carbide burs. Caries removal was performed according to each system, and the operative time was recorded. Residual bacterial contamination was estimated through colony-forming unit counts. Residual dentin was assessed using DIAGNOdent™ laser fluorescence, Vickers microhardness testing, and SEM at multiple magnifications. **Results:** Mean removal times were 2.82 ± 1.64 min for Carisolv™, 2.50 ± 1.15 min for Papacarie^®^, 2.05 ± 0.70 min for Smart Burs™, and 0.61 ± 0.44 min for carbide burs. Residual bacterial growth was detected in all groups. Vickers microhardness values were 79.2 ± 24.66 VHN for Carisolv™, 79.6 ± 22.30 VHN for Papacarie^®^, 83.4 ± 28.73 VHN for Smart Burs™, and 92.9 ± 24.66 VHN for intact dentin control. SEM revealed dentin morphology closer to the control after carbide bur excavation, partially obliterated tubules after Carisolv™, and more evident tubular obliteration after Papacarie^®^ and Smart Burs™. **Conclusions:** Chemomechanical excavation, particularly Carisolv™, generated a residual dentin substrate with microhardness values compatible with a conservative restorative approach, although with longer operative time than conventional burs. The findings support the role of selective caries removal in minimally invasive restorative protocols and highlight the need to connect residual dentin characteristics with adhesive performance, bioactive materials, and long-term restoration outcomes.

## 1. Introduction

Dental caries is a biofilm-mediated, sugar-driven, dynamic disease characterized by alternating phases of demineralization and remineralization of dental hard tissues [1,2]. In restorative dentistry, the management of cavitated carious lesions has therefore shifted from the traditional concept of complete caries excavation toward biologically informed strategies that aim to control the disease process, preserve tooth structure, and maintain pulp vitality [3,4,5,6]. The success of restorative treatment increasingly depends not only on the restorative material placed but also on the biological and structural quality of the residual dentin substrate [3,7,8].

A central principle of minimally invasive caries management is the distinction between infected and affected dentin. Infected dentin is highly contaminated, necrotic or irreversibly denatured, and poorly suitable for remineralization; consequently, it must be removed to allow adequate restoration and lesion control. Affected dentin, in contrast, is partially demineralized but may retain collagen organization and remineralization potential, making its preservation desirable when the substrate is firm and sealable [4,5,6,9]. International consensus recommendations on carious tissue removal support selective removal strategies according to lesion depth and restorative requirements, with the purpose of avoiding unnecessary dentin sacrifice and reducing the risk of pulp exposure [4,5].

Conventional tungsten carbide burs remain a common clinical reference for caries excavation because they are efficient and familiar to clinicians. Nevertheless, rotary instrumentation may remove sound or potentially remineralizable dentin and may contribute to patient discomfort through vibration, pressure, noise, and heat generation [10,11,12]. These limitations are particularly relevant in pediatric dentistry and in patients with dental anxiety, where minimally invasive and atraumatic restorative approaches can improve treatment acceptance [13,14]. For this reason, alternative excavation methods have been developed to promote selective caries removal while preserving the dentin substrate intended to receive adhesive, ionomeric, resin-based, or bioactive restorative materials [7,8,15].

Chemomechanical caries removal systems, such as Carisolv™ and Papacarie^®^, rely on the chemical softening of degraded collagen in carious dentin, followed by mechanical removal with hand instruments [16,17,18]. Carisolv™, which combines sodium hypochlorite with amino acids, has been reported to selectively remove carious dentin while preserving harder dentin [16,19]. Papacarie^®^ is a papain-based gel designed to act on necrotic and partially degraded collagen fibers, with potential advantages related to atraumatic management and pediatric use [18,20]. In parallel, self-limiting polymer burs, such as Smart Burs™, were introduced to remove softened dentin while reducing cutting efficiency when harder dentin is encountered, thereby supporting a conservative mechanical excavation concept [21,22].

From a restorative standpoint, the clinical relevance of a caries removal technique cannot be judged only by the time required for excavation. Residual bacterial load, dentin microhardness, mineral density, smear layer characteristics, dentinal tubule patency, and surface topography may influence substrate conditioning, adhesive infiltration, hybrid layer formation, ion exchange, sealing ability, and the behavior of bioactive restorative materials [7,8,15,23,24,25,26]. Previous investigations have shown that different excavation strategies may alter residual dentin hardness, morphology, mineral density, and bacterial persistence within dentinal tubules [15,17,19,23,24,25,26,27]. Therefore, a multimodal evaluation of the residual dentin substrate is necessary to connect selective caries removal with contemporary restorative caries management.

Artificial caries models are commonly used in comparative in vitro studies because they allow better control of lesion characteristics than naturally carious teeth [15,23,24]. Natural carious lesions vary widely in depth, mineral loss, bacterial composition, dentin hardness, organic matrix degradation, and pulpal proximity, which may introduce substantial variability when comparing excavation methods [1,2,4,5,6,7].

The present in vitro study was designed to compare two chemomechanical systems, Carisolv™ and Papacarie^®^, with two mechanical approaches, Smart Burs™ and conventional carbide burs, using an artificial caries model associated with *Streptococcus mutans* biofilm. The original experimental design included standardized class I cavities in extracted permanent molars, artificial demineralization, biofilm formation, caries removal, colony-forming unit counts, DIAGNOdent™ laser fluorescence, Vickers microhardness testing, and scanning electron microscopy of residual dentin. The aim of the present manuscript was to evaluate whether the excavation method influences caries removal time, residual bacterial contamination, fluorescence values, microhardness, and residual dentin topography, with emphasis on the restorative implications of the resulting dentin substrate.

## 2. Materials and Methods

### 2.1. Study Design

This was an experimental, comparative, in vitro study using extracted permanent molars. The study evaluated four caries removal techniques: Carisolv™ (MediTeam Dental AB, Sävedalen, Sweden), Papacarie^®^ (Fórmula & Ação, São Paulo, Brazil), Smart Burs™ (SS White Dental, Lakewood, NJ, USA), and conventional tungsten carbide burs. The primary restorative endpoint was the quality of residual dentin after excavation, assessed through Vickers microhardness and SEM morphology. Secondary endpoints included caries removal time, residual bacterial load, and DIAGNOdent™ pen 2190 (KaVo Dental GmbH, Biberach, Germany) fluorescence values.

### 2.2. Tooth Selection

Sixty-eight freshly caries-free extracted molars were collected and stored in a 0.2 wt% thymol solution at 4 °C until use. The molars were initially screened under stereomicroscopy (Leica DM IL LED, Leica Microsystems, Wetzlar, Germany) observation. Teeth were included if they presented complete crowns and no extensive structural destruction. Teeth were excluded when they presented crown fracture, extensive caries, restorations, previous endodontic treatment, or structural defects that could interfere with cavity standardization or laboratory processing. After screening, 48 molars fulfilled the selection criteria and were included.

### 2.3. Standardized Cavity Preparation

All samples were fixed in acrylic resin (NicTone; MDC Dental, Guadalajara, Mexico), and each specimen was individually labeled. A mounting jig was used to standardize tooth positioning and align the occlusal surface for cavity preparation. Conventional G. V. Black class I cavities were prepared by a single operator using a high-speed handpiece with spherical #8 carbide burs (SS White Dental, Lakewood, NJ, USA) under continuous cooling to maintain procedural consistency and reduce inter-operator variability. The cavities were standardized to approximately 10 mm in mesiodistal length and 5 mm in depth, reaching dentin. After preparation, the molars were ultrasonically cleaned in distilled water for 5 min using an ultrasonic cleaner (Quantrex, Kearny, NJ, USA). Finally, the external crown surfaces were coated with clear nail varnish to restrict experimental demineralization to the cavity region.

### 2.4. Artificial Caries Model

Artificial caries-like lesions were induced by immersing the specimens in a demineralizing solution containing 1.5 mM CaCl_2_, 0.9 mM KH_2_PO_4_, 150 mM KCl, and 0.1 mM sodium acetate (Sigma-Aldrich, St. Louis, MO, USA), adjusted to pH 4.5 with distilled water [15]. The teeth were incubated at 37 °C under continuous agitation for 240 h. The demineralizing solution was replaced every third day to maintain acidic conditions, as pH changes were observed during incubation. Demineralization was monitored using laser fluorescence diagnosis with DIAGNOdent™ (KaVo Dental GmbH, Biberach, Germany). Mineral changes at the cavity floor were estimated at three standardized points before immersion in the demineralizing solution and after 180 and 240 h of incubation. Three measurements were obtained from the cavity floor. Values were interpreted using the manufacturer’s reference ranges: 0–13, sound tooth; 14–20, superficial enamel caries; 21–29, deep enamel caries; and >30, dentin caries.

A suspension of *Streptococcus mutans* ATCC^®^ 25175™ (American Type Culture Collection, Manassas, VA, USA) adjusted to approximately 0.5 McFarland was prepared and cultured in Mueller–Hinton medium (Becton Dickinson, NJ, USA) under partial anaerobic conditions. Ten microliters of bacterial suspension was placed onto a cotton pellet with culture medium and inserted into each cavity. Cavities were temporarily sealed with IRM ZOE (Dentsply Sirona, Charlotte, NC, USA). IRM ZOE was used as a temporary sealing material to maintain the bacterial inoculum inside the standardized cavity model during incubation. However, because zinc oxide–eugenol materials may release eugenol with potential antimicrobial activity, this factor was considered when interpreting the residual bacterial load results. Specimens were incubated at 37 °C. Every third day, the temporary material was removed, culture medium was replenished, and the cavities were resealed. Biofilm development was maintained for 15 days. A standardized monospecies *S. mutans* biofilm model was selected to provide a reproducible cariogenic challenge and to allow controlled comparison among caries removal methods. However, this model was not intended to reproduce the full ecological complexity or microbial succession of natural carious lesions.

### 2.5. Experimental Groups and Caries Removal Protocols

After biofilm development, specimens were assigned to four groups (n = 15/gp) according to the caries removal method. Carisolv™ group and Papacarie^®^ group: gel was prepared according to the manufacturer’s instructions. Then the gel was applied to the cavity and left in contact with the tissue for 30 s. Softened dentin was removed using sterile dentin excavators (Hu-friedy curette, Chicago, IL, USA). The cavity was cleaned with a moist cotton pellet, and the procedure was repeated until no softened dentin was clinically detected. Smart Burs™ group: Caries removal was performed with acrylic burs mounted on a low-speed contra-angle handpiece, according to manufacturer recommendations. Excavation continued until softened dentin was removed and the bur no longer cut effectively. Conventional carbide bur group: Caries removal was performed using tungsten carbide burs (SS White Dental, Lakewood, NJ, USA) in a low-speed handpiece until a clinically firm dentin surface was reached. A caries detector dye (Viarden, Mexico City, Mexico) was used as an auxiliary visual method to support the identification of residual carious tissue. The removal time was recorded in minutes for each specimen. All procedures were executed in a horizontal laminar chamber flow.

### 2.6. Residual Bacterial Load

Immediately after caries removal, samples were collected from the cavity floor by gently scraping the prepared dentin surface with sterile excavators. Subsequently, 100 µL of Mueller–Hinton broth (Becton Dickinson, Franklin Lakes, NJ, USA) was inoculated into each cavity, gently agitated over the preparation surface, and recollected with a sterile pipette together with the remaining dentin debris. Biofilm collection from the cavity floor was performed by the same trained operator following a standardized scraping and broth recovery protocol. The recovered suspension was transferred into 5 mL of fresh Mueller–Hinton broth and incubated at 37 °C for 24 h. After incubation, aliquots from each sample were vortexed, seeded onto Mueller–Hinton agar plates (Becton Dickinson, Franklin Lakes, NJ, USA), and incubated at 37 °C for 24 and 48 h. Colony-forming units (CFU) were counted and recorded. The presence of viable residual bacteria was interpreted as an indicator of biological control after caries excavation, rather than as a criterion of complete cavity sterilization.

### 2.7. Vickers Microhardness

For Vickers microhardness analysis, five molars from each experimental group were randomly selected. The specimens were sectioned at the cervical level and through the mesial cavity walls, extending slightly above the cavity floor, using a low-speed diamond wheel saw (South Bay Technology Inc., San Clemente, CA, USA) under continuous irrigation until dentin blocks were obtained. The base of each dentin block was sequentially polished with #600, #1000, #1500, and #2000 waterproof abrasive paper (Fuji Star, Sankyo Rikagaku, Okegawa, Japan), taking care not to alter the cavity floor surface. Intact healthy dentin blocks were used as positive controls and processed under the same conditions. Residual dentin hardness was evaluated using a Vickers microhardness tester (DongGuan Sinowon Precision Instruments, Nancheng, DongGuan, China). A diamond indenter was applied to the dentin surface using a 10 N load for 10 s. A total of 50 indentations were performed across the experimental groups, and the values were recorded as Vickers hardness number (VHN).

### 2.8. Scanning Electron Microscopy

Dentin blocks were obtained from the cavity floor after caries excavation to evaluate the micromorphological characteristics of the remaining dentin surface. The specimens were carefully dehydrated, mounted on aluminum stubs, and sputter-coated with a thin layer of gold particles to improve surface conductivity during scanning electron microscopy analysis. The samples were examined using scanning electron microscopy (SEM; JEOL, Akishima, Tokyo, Japan). Micrographs were acquired using secondary electron imaging at 100×, 500×, 1000×, 2000×, and 5000× magnifications, with an accelerating voltage of 20 kV.

### 2.9. Statistical Analysis

All experimental procedures were performed by a single investigator who was blinded to the study groups, while the statistical analysis was conducted independently by another author, following a single-blind design. Data distribution was assessed using the Shapiro–Wilk normality test. Changes in DIAGNOdent™ values during the artificial demineralization protocol were analyzed using the Wilcoxon signed-rank test, since these measurements were obtained repeatedly from the same specimens. Comparisons involving categorical outcomes, including the presence or absence of bacterial growth, were evaluated using McNemar test, Yates’ continuity correction, and odds ratio analysis, as appropriate. Vickers microhardness values among the experimental groups and intact dentin control were analyzed using one-way analysis of variance (ANOVA), followed by Tukey’s post hoc test for multiple comparisons. All statistical analyses were performed using SPSS software, version 25.0 (IBM Corp., Armonk, NY, USA), and statistical significance was set at *p* < 0.05.

## 3. Results

### 3.1. In Vitro Caries Model

Molars exposed to the demineralizing solution showed a progressive increase in DIAGNOdent values above 30 over time. The mean fluorescence values were 55.17 ± 18.68 at baseline, 55.80 ± 28.01 after 120 h, and 63.01 ± 29.92 after 240 h of incubation. The Wilcoxon signed-rank test showed significant changes in the degree of demineralization during the experimental period. When the 240 h values were compared with baseline and 120 h measurements, statistically significant differences were observed (*p* < 0.05 and *p* < 0.001, respectively). In contrast, no significant difference was detected between baseline and 120 h. These findings indicate that measurable demineralization became more evident after 120 h of incubation at 37 °C, with a greater increase observed at 240 h.

### 3.2. Caries Removal Time

The conventional carbide bur group showed the shortest mean removal time (Table 1). The mean removal time was 0.61 ± 0.44 min for carbide burs (*p* < 0.05), 2.05 ± 0.70 min for Smart Burs™, 2.50 ± 1.15 min for Papacarie^®^, and 2.82 ± 1.64 min for Carisolv™. Thus, mechanical rotary excavation was faster than chemomechanical excavation. However, operative speed should be interpreted together with substrate preservation, since time alone does not reflect the biological or restorative quality of residual dentin.

### 3.3. Residual Bacterial Load

Residual bacterial growth was detected after caries removal in all experimental groups, indicating that none of the evaluated techniques completely eliminated viable bacteria from the dentin surface under the conditions of this in vitro model. Overall, CFU values tended to increase over time, particularly at 72 h of incubation. However, only the Carisolv™ group showed a statistically significant difference between pre- and post-excavation values at 72 h, with lower CFU counts after treatment (*p* < 0.05), suggesting a significant reduction in residual bacterial growth at this time point. In contrast, the Papacarie^®^, SmartBurs™, and tungsten carbide bur groups showed residual bacterial recovery, with no statistically significant differences between pre- and post-excavation values (*p* > 0.05). These findings suggest that caries excavation reduces infected tissue but should not be interpreted as complete dentin sterilization; therefore, effective cavity sealing, appropriate restorative materials, and caries risk control remain essential for biological management of the lesion.

### 3.4. DIAGNOdent™ Laser Fluorescence

DIAGNOdent™ values after treatment were higher than those of the sound dentin control in all experimental groups. Mean values were 99.0 ± 0.5 for Carisolv™, 88.3 ± 5.5 for Papacarie^®^, 80.6 ± 17.0 for Smart Burs™, and 70.9 ± 16.8 for carbide burs, whereas the sound dentin control showed markedly lower values of approximately 5.0 ± 3.0 (*p* < 0.05; ANOVA followed by Tukey’s post hoc test). These findings indicate that the residual substrate retained fluorescence characteristics compatible with previously demineralized dentin. In the context of selective caries removal, this does not necessarily indicate clinical failure, since affected dentin may be intentionally preserved when it is firm, non-infected, and suitable for sealing.

### 3.5. Vickers Microhardness of Residual Dentin

The Vickers microhardness of residual dentin was 79.2 ± 24.66 VHN for Carisolv™, 79.6 ± 22.30 VHN for Papacarie^®^, and 83.4 ± 28.73 VHN for Smart Burs™. The tungsten carbide bur group showed the lowest microhardness value, with 60.42 ± 4.40 VHN (*p* < 0.05), whereas the intact dentin control showed a mean value of 92.9 ± 24.66 VHN (Table 2). These results indicate that the tested minimally invasive methods produced residual dentin with microhardness values closer to those of intact dentin compared with conventional rotary excavation. The lower VHN values observed in the carbide bur group suggest a softer residual dentin substrate, which may be associated with the characteristics of the remaining dentin surface after rotary instrumentation.

### 3.6. SEM Surface Morphology

**Figure 1:**
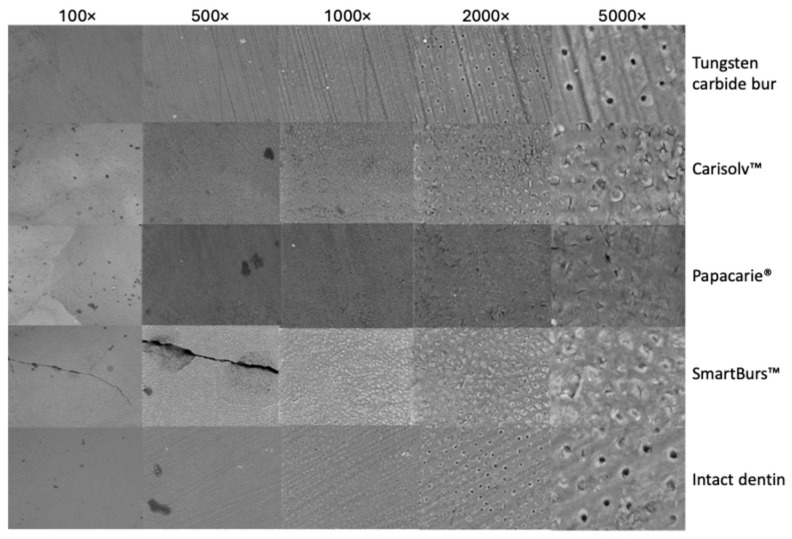
Representative SEM micrographs of residual dentin surfaces after different caries removal methods. Rows correspond to the experimental groups: tungsten carbide bur, Carisolv™, Papacarie^®^, SmartBurs™, and intact dentin control. Columns show increasing magnifications from left to right: 100×, 500×, 1000×, 2000×, and 5000×. The images show method-dependent differences in surface regularity, smear layer distribution, dentinal tubule exposure, and tubular obliteration. Conventional carbide burs produced a more regular surface with visible instrumentation marks and exposed tubules, whereas Carisolv™, Papacarie^®^, and SmartBurs™ showed greater surface irregularity and variable tubule occlusion. SEM: scanning electron microscopy.

SEM analysis revealed method-dependent differences in the micromorphology of the residual dentin surface after caries removal (Figure 1). The conventional tungsten carbide bur group showed a more regular surface pattern, with parallel cutting marks and clearly exposed dentinal tubules, particularly at higher magnifications. This morphology was closer to the intact dentin control, although the bur-treated surface exhibited more evident mechanical instrumentation marks.

In contrast, the Carisolv™ group showed a more irregular residual dentin surface, with partial obliteration of dentinal tubules and areas covered by a smear-like layer. At higher magnifications, the dentin surface appeared heterogeneous, with partially exposed tubules and localized surface deposits. The Papacarie^®^ group presented greater surface irregularity and more evident tubular occlusion, suggesting a residual substrate with increased smear layer formation or organic debris retention after chemomechanical excavation.

The SmartBurs™ group showed a distinct surface pattern characterized by marked irregularity, partial tubular obliteration, and areas of roughened dentin. This morphology is consistent with the selective and self-limiting action of polymer burs, which preferentially remove softer infected dentin while preserving firmer affected dentin. The control dentin showed a comparatively organized surface, with visible dentinal tubules and a more homogeneous morphology.

Overall, these findings suggest that each caries removal method generated a characteristic dentin surface. Conventional carbide burs produced a more regular but mechanically instrumented substrate, whereas chemomechanical methods and SmartBurs™ tended to preserve a more irregular residual dentin surface with variable degrees of tubule occlusion. These micromorphological differences are clinically relevant because dentinal tubule exposure, smear layer distribution, and surface roughness may influence adhesive infiltration, hybrid layer formation, and the interaction of dentin with bioactive, adhesive, or ionomeric restorative materials.

## 4. Discussion

The present study evaluated chemomechanical and mechanical caries-removal methods with respect to residual dentin quality. This approach is consistent with current restorative caries management, where excavation is no longer considered an isolated operative step but rather the first stage of a biologically driven restorative workflow [3,4,5,6,7,8]. The main findings indicate that the evaluated methods differed in operative time and residual dentin morphology, while the Vickers microhardness values obtained after conservative excavation were broadly comparable to those of intact dentin controls. These results support the relevance of selective caries removal as a strategy for preserving a restorable dentin substrate.

Conventional carbide burs showed the shortest removal time. This result agrees with previous reports indicating that rotary excavation is generally faster than chemomechanical methods [10,12,17,21]. However, operative time alone should not be interpreted as a direct indicator of clinical superiority. The goal of minimally invasive caries management is to remove irreversibly infected tissue while preserving remineralizable or structurally useful dentin [4,5,6]. Therefore, a method that is slower but more selective may still be clinically valuable when maintaining pulp vitality, reducing patient discomfort, or preserving residual dentin is a priority [13,14,20].

Carisolv™ and Papacarie^®^ required longer excavation times than conventional carbide burs. This finding is expected because chemomechanical systems depend on chemical softening of carious dentin followed by manual removal [16,17,18]. Previous studies have similarly reported that Carisolv™ may increase treatment time compared with conventional rotary instruments, although it may reduce discomfort and support tissue preservation [16,17,19]. Papain-based systems such as Papacarie^®^ have also been proposed as atraumatic alternatives, particularly in pediatric dentistry, because they can reduce dependence on rotary instrumentation and local anesthesia in selected cases [18,20]. In this context, longer operative time should be balanced against patient-centered outcomes and substrate preservation.

Residual bacterial growth was detected in all groups. This finding reinforces a key principle of contemporary caries management: caries excavation does not necessarily sterilize dentin [24,27]. Bacteria can persist within dentinal tubules after both mechanical and chemomechanical removal, and the biological success of selective caries removal depends on lesion sealing and interruption of nutrient supply rather than complete elimination of all microorganisms [4,5,24]. This interpretation is particularly relevant for restorative procedures using glass ionomer cements, resin-modified glass ionomers, adhesive resin composites, and bioactive materials, in which marginal integrity and internal sealing may be decisive for lesion arrest and prevention of secondary caries [7,8,28].

The Vickers microhardness results suggest that Carisolv™, Papacarie^®^, and Smart Burs™ can leave residual dentin with hardness values approaching those of intact control dentin. This is consistent with previous work showing that conservative or chemomechanical excavation may preserve a mineralized dentin substrate suitable for restoration [15,23,25]. Nonetheless, dentin hardness should not be interpreted as the only determinant of restorative performance. Adhesive behavior is also influenced by collagen integrity, residual mineral content, tubular occlusion, smear layer thickness, moisture, and dentin surface chemistry [7,8,26,29]. Thus, the microhardness data should be integrated with SEM morphology and future bond strength or sealing evaluations.

SEM observations showed method-dependent differences in residual dentin topography. The carbide bur group showed a morphology closer to intact dentin, whereas Carisolv™ produced partially obliterated dentinal tubules and Papacarie^®^ and Smart Burs™ showed more evident tubular obliteration. Previous SEM studies have demonstrated that excavation systems can produce distinct smear-layer patterns and tubular exposure profiles [23,26]. These differences may influence the performance of etch-and-rinse adhesives, self-etch adhesives, universal adhesives, and ionomeric materials. For etch-and-rinse systems, smear layer removal and collagen exposure are critical for hybrid layer formation; for self-etch and universal adhesives, smear layer characteristics and residual mineral availability can influence monomer infiltration and chemical bonding; and for glass ionomer or bioactive systems, residual mineral content may support ion exchange and interfacial maturation [7,8,26,28,29].

The high DIAGNOdent™ values observed after excavation should be interpreted cautiously. Laser fluorescence is useful for detecting demineralized or altered dental tissue, but residual fluorescence does not necessarily mean that all remaining tissue must be removed, particularly under selective caries removal principles [4,5,6]. A firm residual dentin substrate may still show altered fluorescence because of previous demineralization, bacterial metabolites, or changes in optical properties. Future studies should complement laser fluorescence with mineral density assessment using micro-computed tomography, transverse microradiography, Raman spectroscopy, or elemental analysis to better define the residual dentin substrate after minimally invasive excavation [23,25].

The restorative implications of the present findings are relevant. Selective caries removal must be integrated with the subsequent restorative strategy. If residual bacteria and altered tubule morphology remain, the quality of the adhesive seal and the properties of the restorative material become essential. Bioactive restorative materials, fluoride-releasing systems, calcium- or phosphate-containing materials, and nanostructured restorative approaches may be particularly useful when residual dentin is preserved intentionally [7,8,28,29]. Therefore, the excavation method should be selected not only based on clinical access and operative time but also on the planned restorative material, lesion depth, pulp risk, patient cooperation, and caries risk profile.

This study has limitations. First, the experiment was performed in vitro, and artificial caries and *S. mutans* biofilm models cannot reproduce the full complexity of natural carious lesions, saliva, dentinal fluid, pulpal pressure, multispecies biofilms, dietary fluctuations, and host factors [1,2]. Second, CFU counting detects cultivable microorganisms but does not capture the complete microbiome or intratubular bacterial distribution [24,27]. Third, the number of teeth used for some subgroup analyses was limited, and repeated measurements within the same tooth should ideally be analyzed using hierarchical or mixed statistical models. Fourth, the study did not assess adhesive bond strength, nanoleakage, restoration survival, or remineralization after restorative placement. These outcomes should be incorporated in future studies to directly link excavation-induced dentin changes with restorative performance.

Despite these limitations, the present work contributes to the field by connecting caries excavation with restorative substrate quality. The combination of operative time, bacterial analysis, fluorescence, microhardness, and SEM morphology provides a more complete understanding of how different caries removal methods condition the residual dentin substrate. This is directly aligned with minimally invasive restorative dentistry and with current priorities in caries management, where preservation, sealing, bioactivity, and long-term restoration success are considered interdependent components of treatment [3,4,5,6,7,8,28].

Another limitation is the use of IRM ZOE as a temporary sealing material during biofilm development. Although it allowed cavity sealing and retention of the bacterial inoculum within the artificial caries model, the possible antimicrobial effect of eugenol release was not independently evaluated. Therefore, ZOE may have influenced biofilm viability or residual bacterial recovery. Future studies should use eugenol-free temporary sealing materials or include an additional ZOE/eugenol control group to distinguish the effect of the sealing material from that of the caries removal method.

Cavity preparation and biofilm collection were performed by a single operator. Although this approach was used to reduce inter-operator variability and standardize the experimental procedures, future studies should include multiple calibrated operators and inter-operator reliability assessment to improve reproducibility and external validity.

The clinical and translational relevance stated that the selective caries removal should be interpreted as a restorative strategy rather than a simple excavation technique. The results suggest that chemomechanical methods may be useful when tissue preservation, patient comfort, and conservative management are priorities. For pediatric dentistry and minimally invasive restorative care, such methods may reduce operative aggressiveness and support preservation of dentin capable of remineralization.

However, residual bacteria and altered dentin morphology indicate that excavation must be paired with appropriate restorative sealing. The clinical success of these approaches likely depends on the complete restorative workflow: lesion diagnosis, selective removal, substrate conditioning, adhesive or ionomeric strategy, restorative material selection, and caries risk management.

## 5. Conclusions

Within the limitations of this in vitro study, chemomechanical and conservative mechanical caries removal methods produced residual dentin with microhardness values approaching intact dentin, although they differed in operative time and SEM morphology. Conventional carbide burs were the fastest method, while Carisolv™ and Papacarie^®^ required longer excavation times but supported a tissue-preserving rationale. Residual bacterial growth was detected in all groups, reinforcing that selective caries removal must be followed by effective sealing and restorative management.

These findings support the integration of selective caries removal into minimally invasive restorative protocols. Future studies should evaluate adhesive performance, bioactive restorative material interactions, marginal sealing, remineralization potential, and long-term outcomes using residual dentin substrates generated by different excavation methods. In addition, multispecies, saliva-derived, or microcosm biofilm models should be incorporated to better reproduce the microbial succession, ecological complexity, and clinical behavior of natural dental caries.

## Figures and Tables

**Table 1:** Caries removal time by excavation method.

Group	Removal Method	Time ± SD (min)	Restorative Interpretation
Carisolv™	Chemomechanical	2.82 ± 1.64	Longer time, tissue-preserving rationale
Papacarie^®^	Chemomechanical	2.50 ± 1.15	Longer time, enzymatic selective approach
Smart Burs™	Self-limiting mechanical	2.05 ± 0.70	Intermediate time, conservative mechanical removal
Carbide burs	Conventional mechanical	0.61 ± 0.44 (*p* < 0.05)	Shortest time, conventional clinical reference

**Table 2:** Vickers microhardness of residual dentin.

Group	Mean ± SD (VHN)	Interpretation for Restorative Substrate
Control dentin	92.9 ± 24.66	Reference intact substrate
Carisolv™	79.2 ± 24.66	Conservative residual dentin compatible with selective excavation
Papacarie^®^	79.6 ± 22.30	Similar hardness to Carisolv™, with different surface morphology
Smart Burs™	83.4 ± 28.73	Similar residual hardness with self-limiting mechanical action
Carbide burs	60.42 ± 4.40	Lowest residual hardness, suggesting a softer dentin substrate after conventional rotary excavation

## Data Availability

The datasets generated and analyzed during the current study are available from the corresponding author upon reasonable request.
